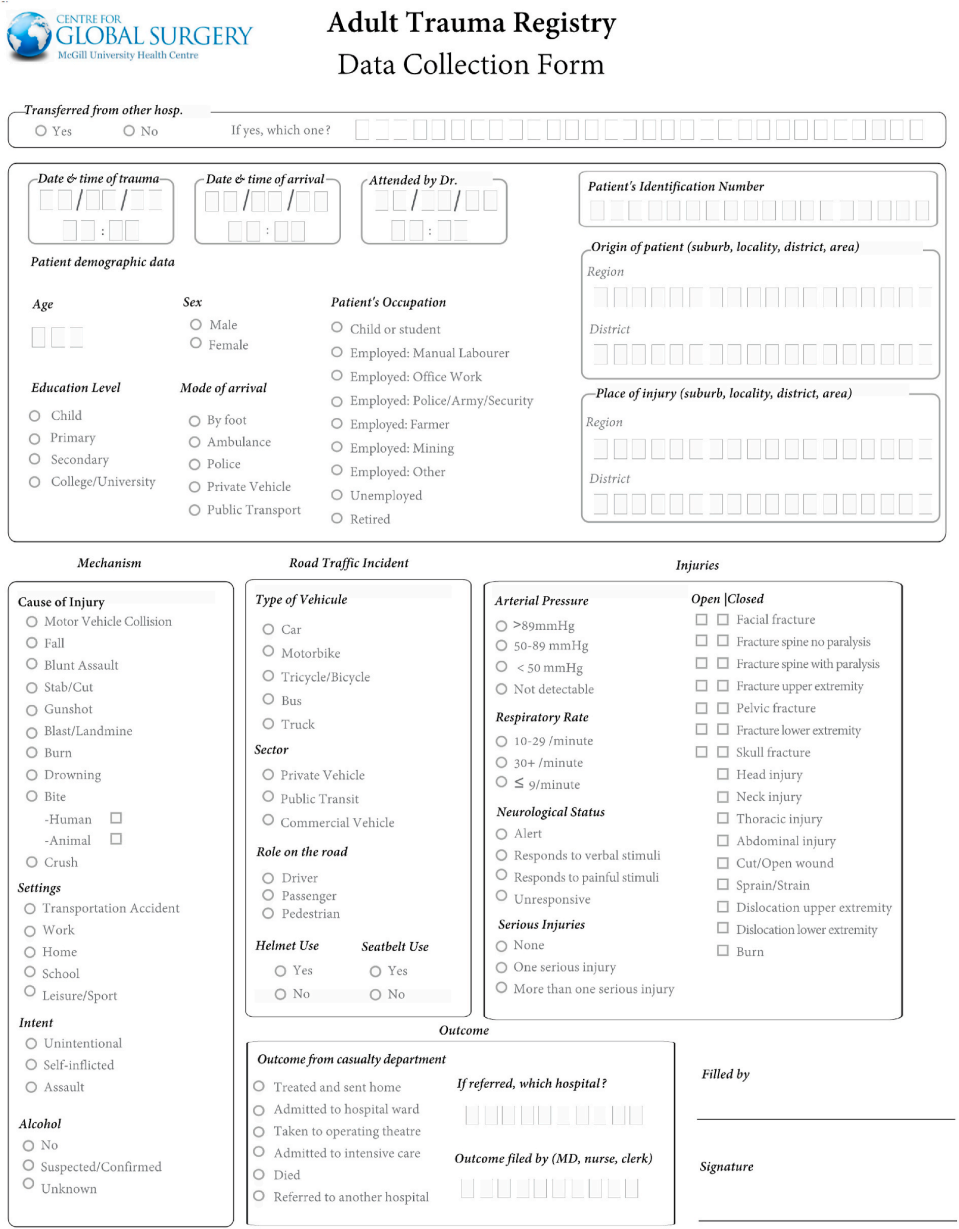# A pilot trauma registry in Peshawar, Pakistan – A roadmap to decreasing the burden of injury – Quality improvement study

**DOI:** 10.1016/j.amsu.2021.103137

**Published:** 2021-12-04

**Authors:** Omaid Tanoli, Hamza Ahmad, Haider Khan, Farhad Ali Khattak, Awais Khan, Alexandre Mikhail, Dan Deckelbaum, Tarek Razek

**Affiliations:** a McGill University Health Centre, Centre for Global Surgery, Montreal, Qc, Canada; b University of Toronto, Department of General Surgery, Toronto, On, Canada; c Bacha Khan Medical College, Mardan, Khyber Pakhtunkhwa, Pakistan; d Khyber College of Dentistry, Peshawar, Khyber Pakhtunkhwa, Pakistan; e Khyber Medical College, Peshawar, Khyber Pakhtunkhwa, Pakistan

**Keywords:** Trauma, Trauma registry, Low- and middle-income countries, Injury surveillance

## Abstract

**Background/local problem:**

In Pakistan, trauma is a significant public health issue accounting for the second leading cause of disability and fifth for healthy years of life lost. Well-developed trauma systems, utilizing trauma registries, have been proven to decrease morbidity and mortality from injuries, and helped to reduce the number of injured patients. In Pakistan, most data on injury are acquired through methods that are retrospective, incomplete, and open to recall bias. To that end, a trauma registry was piloted at the Lady Reading Hospital (LRH) in Peshawar, Pakistan to elucidate the importance of trauma registries in designing healthcare targeted quality improvement initiatives.

**Intervention:**

Upon receiving ethics approval, a twenty-five-point registry was piloted at the Lady Reading Hospital.

**Methods:**

The pilot implementation was carried out from May 9th to May 13th, 2018.

**Results:**

A total of 267 patients were included in the pilot registry. Motor vehicle collisions were the most prevalent cause of injury (46%). The other causes of injury included falls (24%), blunt assaults (9%), stabs/cuts (8%), gunshots (6%), crush injuries (3%), burns (2%), and blasts/landmines (2%). Most patients were treated in the trauma bay and required no further acute intervention (51%).

**Conclusion:**

This 5-day pilot trauma registry was the first of its kind in Peshawar, Pakistan, and despite its short course, an immense amount of data was garnered on the epidemiology of injury in the region. Significantly, the data collected can already be used to develop evidence-based changes, which will effectively minimize the impact of trauma.

## Highlights

• 267 patients were captured over the course of 5 days.
• Motor vehicle accidents were the most prevalent cause of injury.
• Private ambulances were the most common form of transportation.
• Registry accurately described the epidemiology of injury in the region.
• Study displays the feasibility of a long-term registry in Northwestern Pakistan.

## 1 Introduction

Injuries are one of the leading causes of morbidity and mortality worldwide, accounting for approximately 1 in 10 deaths. The burden of trauma is disproportionately centered on low- and middle-income countries (LMICs) [1], including Pakistan, where trauma is a significant public health issue as it accounts for the second leading cause of disability, fifth for healthy years of life lost, and eleventh for premature death [2]. In order to progress towards the goals outlined in the Lancet Commission on Global Surgery; reducing the number and improving the care injured patients receive, it is of the utmost significance to implement trauma registries across Pakistan to gain vital information regarding the current situation of trauma care.

Well-developed trauma systems have been proven to not only decrease morbidity and mortality from injuries, they have helped to reduce the number of injured patients as well [3]. A key feature of trauma systems is the trauma registry. In its most basic form, it is a data-acquisition tool, which can accurately and prospectively collect epidemiological data on injury. However, they are essential for quality improvement and public health interventions in order to both reduce the number of injured patients and the morbidity and mortality associated with trauma [4]. Various registries have been implemented across LMICs in order to achieve these goals. Even though, variables within different trauma registries differ in various contexts, the majority include data pertaining to important factors such as pre-hospital care, physiological response (e.g., vital signs, laboratory data), in-hospital interventions, injury classification, complications, and patient outcomes [5].

In Pakistan, the vast majority of data on injury is acquired through administrative data, police reports, and surveys – all of which are retrospective, incomplete, and are open to recall bias [2]. This hinders governmental and non-governmental organizations from implementing evidence-based interventions in order to reduce the burden of injury in Pakistan. The only previous trauma registry implementation was carried out at Aga Khan University Hospital in Karachi, Pakistan in which they were able to elucidate important variables, which could be used for quality improvement [6]. However, Pakistan is the 5th most populous country in the world and with an ever-growing younger population, more data is required in order to reduce the burden of injury within the nation.

To that end, a trauma registry was piloted at the Lady Reading Hospital (LRH) in Peshawar, Pakistan in the Khyber Pakhtunkhwa province to elucidate the importance of trauma registries in designing targeted quality improvement initiatives including; planning resource allocation, understanding pre-hospital care and transport priorities, and tracking changes in trauma system performance over time [7].

## 2 Methods

### 2.1 Study setting

The Lady Reading Hospital (LRH) is located in Peshawar, the largest city in the province of Khyber Pakhtunkhwa, with an estimated population of 2 million people. It has a 30-bed accident and emergency department with 10 operating rooms dedicated to emergency surgeries. It acts as the major trauma referral center for the province of Khyber Pakhtunkhwa with an estimated catchment population of 35 million people. Given its significance as a trauma center, it was the ideal location to pilot a single institution trauma registry in the region.

### 2.2 Intervention

The trauma registry used was developed by the MUHC – Centre for Global Surgery, which had previously been successfully piloted in other LMICs including Mozambique and Tanzania [8]. Permission for use was obtained from the MUHC. The trauma registry is a twenty-five-point registry which included administrative data, geographical information, basic patient information, mechanism of injury, specific road traffic incident data points, basic physiological information, and outcomes as shown in Appendix A.

### 2.3 Data collection

The pilot implementation was carried out from Wednesday May 9th to Tuesday, May 13th, 2018. Recommendations outlined by Porgo, Moore and Tardif [5] were followed to ensure data quality, which included measures for data completeness, accuracy, precision, and timeliness. An onsite researcher was positioned in the trauma bay from 0700 to 2100 daily and filled out the trauma registry form for each trauma arriving in the department for accuracy. This was initially filled out on paper, and following completion of data collection, it was later transcribed to an electronic database to ensure the precision of the records. For each patient, there was an attempt to collect every data-point at the time of presentation or shortly after initial resuscitation to ensure completeness and timeliness. There were no specific exclusion criteria, however, in order to be included in the trauma registry, patients had to have injuries severe enough to present or be transferred to the trauma bay at LRH.

### 2.4 Data completeness

While the data collector was on-site, there was no patient missed from the registry. The identification numbers of 36% patients were not available and 3 of the referred patients did not have the name of the referring hospital. In the demographics section, many patients were missing the variables “origin of patient” and “location of injury”. No other variables had missing information.

### 2.5 Data analysis

The trauma registry data was initially assessed for completeness. The online registry database developed automated descriptive statistics allowing for analysis of the variables. Moreover, data from the registry was used to determine the Kampala Trauma Score (KTS) [9] for each individual patient. Finally, the work has been reported in line with the Standards for Quality Improvement Reporting Excellence (SQUIRE) criteria [10].

## 2.5 Ethics approval

Ethics approval was received from the Hospital Director and Head of the Accident & Emergency Department in accordance with the regulations at LRH.

## 3 Results

### 3.1 Demographics

A total of 267 patients were included in the pilot registry at the end of data collection. This comprised a total of 71% males and 29% females with a mean age of 23.2 years (SD 17 years). The majority of patients were from Peshawar (64%), while the most common regions of injury were Peshawar (64%), Greater Khyber Pakhtunkhwa (13%), and the Federally Administered Tribal Areas (2%).

A majority of patients had a primary level of education or none (54%), while 14% had a secondary level of education, and 9% had a college degree or higher. 23% of patients were children who had not initiated school yet. In terms of occupation, 11% worked in manual labor, 2% were in the police or army, 0.5% had an office job, 0.5% were farmers, 25% had an occupation not specifically listed on the registry, 3% were retired, 14% were unemployed, and 44% were either children or students.

### 3.2 Transportation and referrals

22% patients were transferred from a referring hospital, with 4% from Mardan Medical Complex (MMC) and 1% from Khyber Teaching Hospital (KTH). The rest were transferred from various District Headquarter Hospitals (DHQs) and Tehsil Headquarter Hospitals (THQs). Transport to the hospital was achieved via ambulance in 32% of patients, 32% in private vehicles, 29% used public transport, 6% arrived on foot, and 1% were brought in by the police.

### 3.3 Injury patterns

**Fig. 1:**
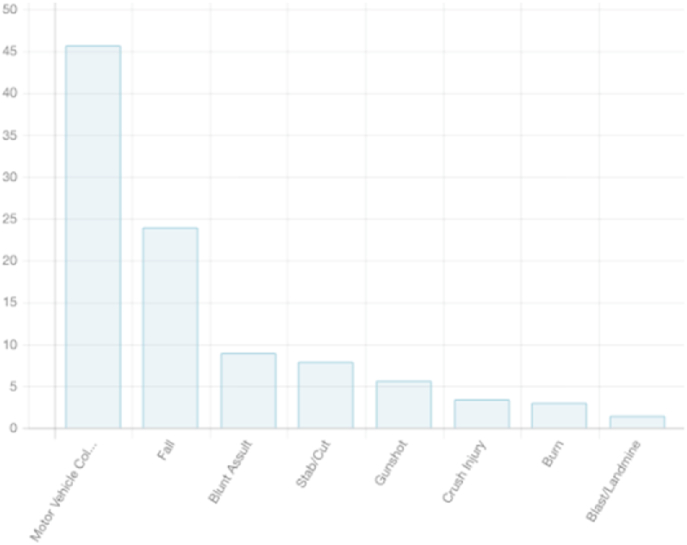
Causes of injury.

Motor vehicle collisions were the most prevalent cause of injury (46%). The other causes of injury are represented in Fig. 1. Of the total injuries, 13% were assaults and 0.5% were self-inflicted. The settings of the various injuries included transportation accidents (46%), at home (33%), workplace injuries (11%), leisure/sport (8%), and at school (3%).

Of the total 267 patients, there were 390 distinct injury patterns. The various types of injuries included cuts/open wounds (37%), head injuries (28%), sprains/strains (7%), closed fractures of the lower extremities (7%) and upper extremities (5%), thoracic injuries (3%), abdominal injuries (3%), burns (2%), open fractures of the lower extremities (2%) and upper extremities (2%), and miscellaneous injuries (4%).

### 3.4 Motor vehicle collisions

**Fig. 2:**
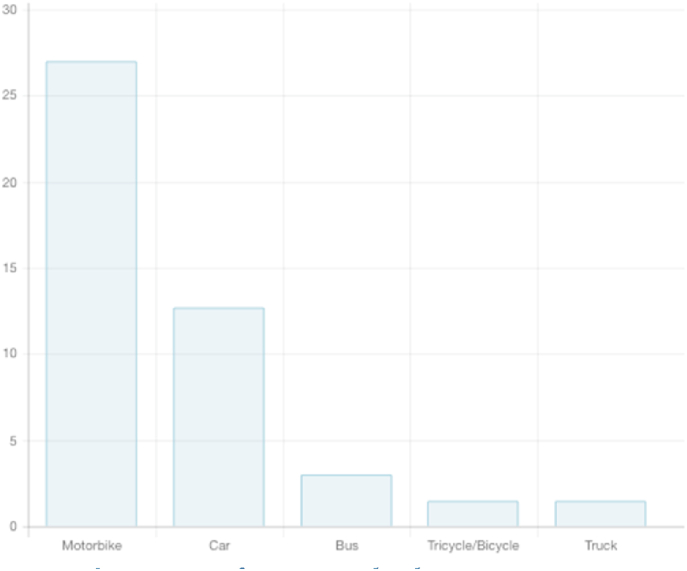
Types of injury vehicle.

There was a total of 122 patients involved in motor vehicle collisions. The types of vehicles were, as represented in Fig. 2. Public transport accidents made up 29% of injuries, while commercial vehicle accidents made up 5% of the injuries. Pedestrians were the most commonly injured patients involved in motor vehicle collisions (45%). Additionally, 31% were the passengers and 24% were the drivers.

Importantly, only 5% patients involved in motorbike and bicycle accidents wore a helmet. Moreover, out of all the patients involved in bus, car, and truck accidents none were wearing a seatbelt at the time of injury.

### 3.5 Injury severity and outcomes

The trauma registry included four variables to measure injury severity including systolic blood pressure, respiratory rate, neurological status, and a measure of seriousness defined by an injury requiring admission or surgical intervention. The vast majority of patients (95%) had a blood pressure greater than 89 mmHg. Moreover, 95% of patients had a respiratory rate between 10 and 29 breaths/minute, 3% had a rate greater than 29 breaths/minute, and 2% had less than 10 breaths per minute. For neurological status, 89% of patients were alert on arrival, 6% responded to verbal stimuli, 3% responded to painful stimuli, and 2% were unresponsive. 7% patients had more than one serious injury, 42% had one serious injury, and 51% had no serious injury. The average Kampala Trauma Score was 14.9 (SD 1.4), indicating most patients did not have very serious injuries.

The majority of patients were treated in the trauma bay and required no further acute intervention 51% These patients had an average KTS score of 15.8. However, 45% of the patients required admission for further treatment including surgical interventions, with an average KTS score of 14.3.2% of patients were taken directly to the operating room (average KTS of 13.5) and 2% of patients died in the trauma bay (average KTS score of 8.8).

### 3.6 Data completeness

During the period in which the data collector was on site, no patients were missed. However, in the recording of variables in the registry, the identification numbers of 36% of patients were not recorded on the registry. Moreover, 21% and 16% of patients were missing the variables “origin of patient” and “location of injury” respectively. Also, 1% of the referred patients did not have the name of their referring hospital on their registry sheets. All other variables were completed for every patient in the pilot registry. Each form took on average 2 min to complete.

## 4 Discussion

The pilot trauma registry at the Lady Reading Hospital in Peshawar, Pakistan was the first attempt at prospective trauma data collection in the region. In total, 267 patients were captured over the course of 5 days, with near-complete forms completed for each patient. Of note, a single registrar was able to effectively capture all patients presenting to the trauma bay throughout the period of this study. Currently, the LRH does have registrars stationed in the trauma bay at all times, however, they are recording only demographic information for patients. Training these registrars to collect information for the trauma registry would be minimal as displayed by the experience of previous trauma registries employed in other LMICs [11,12]. In terms of entering data into the online registry, this took on average 1 min per patient, however, with registrars inputting data directly into the online database, this would minimize the need for extra personnel and eradicate this superfluous step in the process. Overall, this speaks to the feasibility of such a project in the long-term and the data collected provides important insight into the epidemiology of injury in the region. An institutionalized trauma registry would provide the necessary information to facilitate evidence-based interventions to curb the growing epidemic of trauma morbidity and mortality in the region. Given the rising number of disability-adjusted life years (DALYs) and years of life lost (YLL) due to injury, such a project is a necessity.

In Pakistan, motor vehicle collisions (MVCs) are the 18th leading cause of death, with an estimated 23,445 deaths from MVCs annually [13]. In keeping with this, MVCs were the most prevalent cause of injury captured in the registry. Of significance, no patient involved in a MVC was wearing a seatbelt at the time of injury, and of the total motorbike/bicycle injuries only 5% of patients were wearing a helmet. Although Pakistan does have a national seat belt and motorcycle helmet law, their enforcement is quite complacent despite being proven to decrease the morbidity and mortality associated with MVCs [14]. The data captured in this pilot registry reaffirms the previous held notions regarding MVCs in Pakistan. Interestingly, pedestrians were the most commonly injured patients from MVCs. In general, out of pocket expenditure for pedestrians involved in MVCs is significantly higher than the average MVC patient [15]. They also represent a so-called low hanging fruit with regards to public health policy. Clearly, more road-side safety is required in order to decrease the number of pedestrians being struck by vehicles. A concerted effort by both government and other stakeholders would work to minimize the number of pedestrians involved in MVCs.

Following motor vehicle collisions, the next most prevalent cause of injury identified were falls. Given the limited amount of snowfall in the region, this is an alarming number. This argues that most of these falls were from height either at home or in the workplace. Most houses in the region are designed with flat rooftops where children commonly spend their leisure time. With such a high number of injuries secondary to falls, it is necessary to implement greater safety mechanisms at home and in the workplace in order to prevent such injuries. Alternatively, with the creation of safe recreational facilities such as parks and recreation centers, children would potentially spend less time playing on their roofs. More work has to be done in order to identify the root cause for the large number of fall injuries in order to minimize this preventable cause of morbidity and mortality in the region.

Private ambulances, public transport, and private vehicles were the most common form of transportation for trauma patients arriving at the LRH. In an optimal pre-hospital system, all seriously injured patients should be transported via ambulance with trained paramedics. In LMICs with limited resources, this is not always possible. However, in order to fill this treatment gap, first-responder layperson training has led to considerable improvements in pre-hospital care. For example, in Uganda, motorcycle taxi drivers were given training in first-responder care, and improvements were noted in bleeding control, airway and breathing management, recovery position, scene management, and safe patient transport [16]. Moreover, in a previous study from Pakistan, an estimated 58% of trauma patients died prior to hospital arrival [17]. Although this estimate may be on the higher side, it is clear that without a structured and organized pre-hospital system, trauma patients’ care will remain sub-optimal. A greater emphasis needs to be placed on the training and development of a paramedic service in the region. However, given the large proportion of patients being transported via public transport/private ambulances and the limited resources, first-responder training could be provided to public transport custodians and private ambulance technicians in order to minimize the detrimental effects of unsafe transport.

The global burden of disease profile for Pakistan lists MVCs as the 13th leading cause of YLL, along with other forms of injury as the 15th, 16th, 18th, 20th, 21st, and 25th leading causes. All together these forms of injury would be the 4th leading cause of YLL in Pakistan, following lower respiratory infections, neonatal encephalopathy, and diarrheal disease [18]. Despite these alarming numbers, there has not been an effort to curb the growing number of injuries. In fact, there has been a 50–100% increase in the DALYs for road traffic injuries, falls, and self-harm from 1990 to 2010 [18]. Moreover, the economic impact of injuries is also staggering as the cost of road traffic injuries alone in Pakistan is estimated at 1.3% of the GDP, or USD 1.6 billion [19]. Despite the massive impact on society economically and individually, the true incidence of injury is likely being under-estimated given the lack of reliable reporting and under-reporting of certain injuries such as workplace accidents [20]. In order to battle this epidemic, the first step is to gather accurate prospective data regarding the epidemiology of injury in different regions. This will allow stakeholders to develop policies, which will effectively reduce the burden of injury in the region.

This 5-day pilot trauma registry was the first of its kind in Peshawar, Pakistan, and despite its short course, an immense amount of data was garnered on the epidemiology of injury in the region. Significantly, the data collected can already be used to develop evidence-based changes, which will effectively minimize the impact of trauma. However, given the limited time period of pilot registry implementations, caution should be taken when extrapolating the data, as the data is open to sampling bias. However, as has been shown in the past, surgical and trauma care interventions are extremely cost-efficient [21]. A long-term trauma registry in the region will inevitably work towards providing the means to reduce the massive burden of injury in Pakistan. Moreover, following the implementation of interventions, a long-term registry could be used to track the effectiveness of said interventions. This pilot registry has just scratched the surface with regards to the utility and impact of a long-term data collection tool. With the ever-growing injury epidemic, it is vital that local stakeholders take steps to develop and implement a long-term registry into the healthcare infrastructure in Peshawar, Pakistan.

## 5 Going forward

Healthcare data acquisition is one of the key principles for quality improvement and to bridge the gap in healthcare disparities between healthcare systems. Data acquisition tools are low-cost interventions that can have extremely large impacts on the quality of care delivered. Most can be programmed to point of care, and can be used on smartphones and tablets, making the process even simpler. In one study, 80% of healthcare workers in LMICs used smartphones or tablets in the workplace [22]. The fact that most useful data acquisition in LMICs only occurs in a limited setting to a directed goal is an indictment on the global healthcare community. As this study illustrates – the potential is there – with limited resources in a harsh environment, it is extremely feasible to gather a large amount of useful data that can be used towards improving care delivered in LMICs. Moving forward, healthcare data acquisition needs to become a fundamental aspect of all healthcare systems, as it will enable healthcare systems in LMICs to improve care, guide goal-directed interventions, and reduce the healthcare gap between LMICs and HICs.

## Please state whether ethical approval was given, by whom and the relevant Judgement’s reference number

Ethical approval received from Head of Accident and Emergency Department at Lady Reading Hospital and Hospital Director. They can be reached at 091–9211430.

## Funding

This research did not receive any specific grant from funding agencies in the public, commercial, or not-for-profit sectors.

## Author contribution

Omaid Tanoli – Study design, data collection, analysis, writing. Hamza Ahmed – Data analysis, writing. Haider Khan – Study design, Data collection. Farhad Ali – Study design, Data collection. Awais Khan – Data collection. Alexandre Mikhail – Writing. Dan Deckelbaum – Study design. Tarek Razek – Study design.

## Provenance and peer review

Not commissioned, externally peer-reviewed.

## Please state any conflicts of interest

None.

## Please state any sources of funding for your research

None.

## Research registration Unique Identifying number (UIN)

1. Name of the registry: Research Registry
2. Unique Identifying number or registration ID: researchregistry6996
3. Hyperlink to your specific registration (must be publicly accessible and will be checked): https://www.researchregistry.com/browse-the-registry#home/registrationdetails/60fb082bf75921001eb6f025/

## Guarantor

Omaid Tanoli.

## Declaration of competing interest

None.

## Figures and Tables

**Figure:**